# Supplementary material for: Writing in the air: Facilitative effects of finger writing in older adults
Source: PLoS One. 2019 Dec 27;14(12):e0226832. doi: 10.1371/journal.pone.0226832 (PMC6934278; doi:10.1371/journal.pone.0226832)
Supplement: Attachment — Submitted filename: Kusho_older_adults_Responses_to_Reviewers_20190819.docx [file pone.0226832.s002.docx]

Dear Editor and Reviewers,

We greatly appreciate your rigorous and fair review of the manuscript as well as your productive comments. The revised manuscript has been modified, mainly with the following points, in response to the comments:

1) We modified the term “cognitive processing” in our conclusion and use more limited language.
2) We added explanations about the participants, the procedure of the kanji construction task, and the stimuli.
3) Several paragraphs were modified to be more readable in terms of paragraph writing.
4) We have added discussions on MMSE/MCI, cognitive reserve, and vocabulary related to kūsho effect.

All comments from the reviewers have been responded to, in turn, on the following pages. In the revised manuscript, revisions are highlighted in red.

We look forward to hearing from you regarding our submission. We would be glad to respond to any further questions and comments that you may have.

Sincerely,

Yoshihiro Itaguchi

Responses to Reviewer 1’s comments	p. 2

Responses to Reviewer 2’s comments	p. 8

Responses to Reviewer 3’s comments	p. 13

**Reviewer #1:**

(1) I would suggest that the abstract be revised to include greater specification of the meaning of the terms ‘kūsho’ and ‘the kūsho effect’. I showed this abstract to several colleagues who have no exposure to kanji cultures. They were wholly mystified as to the meaning of ‘writing in the air or on a surface with one’s index finger’. Unless you anticipate addressing only a readership that is already familiar with kūsho, I think you need to add to the Abstract some further identification of its meaning.

Authors’ response

First, we greatly appreciate Reviewer #1’s extensive and valuable comments. The reviewer pointed out that the abstract lacks a clear explanation of “kūsho” and “the kūsho effect” for readers who are not familiar with the phenomenon. We agree with this point and we have added a detailed definition of the terms in the abstract of the revised manuscript, as follows: *“Kūsho, which refers to a behavior in which one moves the index finger as a substitute for a pen in the air or on a surface, mostly used when trying to recall the shape of a written character or the spelling of a word, has been known to facilitate cognitive task performance among kanji writing-system users” (L. 17).*

(2) Line 66: ‘Individuals with demographic factors related to cognitive decline’ is both vague and disguises an inference you are making. Can you be more straightforward? How about ‘older adults, among whom we assume cognitive decline is underway’?

Authors’ response

We are sorry that the expression was confusing. In the revised manuscript, we have modified the sentence as follows: *“**We thus tentatively hypothesized that individuals among whom we assume cognitive decline is underway would show larger kūsho effects”* (L. 70).

(3) Line 67: This raises a major problem: from here on out you refer repeatedly to ‘larger [/ smaller] kūsho effects’. You don’t actually measure the SIZE of the effect, but rather only the frequency of its presence / absence. This comes to a head in the interpretation of Figure 3b, where the horizontal axis records the kūsho effect from –6 to (+)6. Presumably, this is not a measure of its ‘larger / smaller’ SIZE, but rather its frequency of use—correct? This needs to be clearly specified. (I would add that Figure 3 is out of focus and difficult to read. Can a new version of it be created, with higher resolution?)

Authors’ response

We are afraid that there has been a misunderstanding about the analysis and the experimental design. First, we evaluated the *size* of the kūsho effect. As was already mentioned in the previous version of the manuscript, we compared the number of correct responses among three experimental conditions (see Fig. 2). Second, we did not count the frequency of the kūsho occurrence in the main experiment; we did so only in the practice trials, and we could not do so in the main experiment because we manipulated the hand condition. Third, as explained in “Materials and methods,” we defined the kūsho effect as the difference in the number of correct responses between the kūsho and static conditions.

In addition, to see the original resolution figures, please click the link above the page that includes a figure (“Click here to access/download;Figure;Figure 1.tif”). We apologize for the confusion.

(4) Line 82. Please provide full details on the content of the MMSE.

Authors’ response

The Mini-Mental Status Exam (MMSE) is a common international cognitive screening test. We have added a brief explanation of the scale in the methods section of the revised manuscript. “*An MMSE, which is a 30-point questionnaire that is used in clinical and research settings to evaluate cognitive impairment, was conducted before…”* (L. 87).

(5) Lines 83-5. Please also explain how you ‘carefully assessed whether participants had sufficient cognitive capacity…’

Authors’ response

During the experiments and conversations, we checked the rationality of the participants’ verbal responses and the appropriateness of their behavior. Additionally, we asked the clinical staff in the citizen center from where we recruited the participants about their daily behavior and intellectual activity. We have added an explanation in the methods section of the revised manuscript as follows: *“…we carefully assessed whether participants had sufficient cognitive capacity to understand and follow the instructions for the experiment by checking the rationality of their verbal responses and the appropriateness of their behavior in the experiment and conversation*” (L. 91).

(6) The authors declare under ‘Data availability’ that ‘All relevant data are within the manuscript and its Supporting Information files.’ However, I would like to see the full set of 60 experimental stimuli, and do not find them in the materials made available to me in the course of this review.

Authors’ response

We are sorry that we were unclear regarding the experimental stimuli. All of the data are available in our previous work (Itaguchi et al. 2017), and we have added them as Supporting information.

(7) Line 16 mentions that ‘This type of puzzle is popular in Japan’. Could you elaborate? If kanji-assembly tasks constitute a familiar puzzle or pastime, how is that carried out? Does kusho play any role in it, a role that might carry over into your results?

Authors’ response

One type of kanji construction task is sometimes used in TV programs. In such tasks, there could be four or more sub-parts for a kanji, but the task itself is the same as ours. In those situations, there are no instructions about finger movements. Thus, kūsho behavior either spontaneously occurs or it does not. Although we do not know the participants’ experiences with this type of quiz, there are no theoretical reasons for the experience to influence the kūsho effect. To clearly explain the situation and why Japanese people are familiar with this task, we have added “…for example, they sometimes appear in TV programs, albeit without any instructions on hand or finger movements…” (L. 116) to the revised manuscript.

(8) Lines 130–1. How, exactly, did you ‘encourage [participants] to turn their eyes from the screen to their right index finger’? Did any participants resist doing so, fail to do so, or fail to maintain their gaze on the kūsho-producing hand?

Authors’ response

Since people do not always watch their fingers when they are doing kūsho, we gave them a clear instruction to watch their finger movements. We have modified the text and added an explanation, as follows: *“…participants were instructed to turn their face from the screen and watch their right index finger or hand in a trial, since people do not always watch their finger when they are doing kūsho. If they turned their eyes away from the finger, one of the experimenters gave an instruction to watch the finger”* (L. 145).

(9) Discussion section. I think this section would benefit for a more conservative statement of your findings. For example, have you really ‘confirmed that kūsho behavior improved cognitive task performance’ as opposed to ‘confirmed that kūsho behavior co-occurs with improved cognitive task performance, relative to performance under the other two test conditions’? Likewise, have you shown that ‘kūsho behavior has a beneficial influence on cognitive processing’ as opposed to that ‘kūsho behavior correlates with greater success at integrating parts of kanji into familiar whole orthographic units’?

Similarly, in lines 330–2, the authors claim that because of the negative relation between kūsho and years of education, ‘our findings…suggest that kūsho is a helpful tool, especially for individuals with declining cognitive function’. I’m not sure you can draw that inference, rather than simply that ‘kūsho is less frequently employed by older individuals with more years of education relative to their age-mates with less education’. I don’t think you’ve shown that less education correlates with greater cognitive decline.

Authors’ response

First, as the reviewer pointed out, our conclusion was a bit overgeneralized. We modified “cognitive task/processing” to use language with a more limited application. “*This study confirms the benefits of finger movement for solving cognitive tasks involving visual processing of written language among older adults and suggests the kūsho effect may be mediated by education*” (abstract, L. 26), “…*we confirmed that kūsho behavior improved cognitive task performance that required visual processing of written language…*” in the first paragraph of the discussion (L. 253), and “…*consistent with the previously proposed idea that kūsho behavior has a beneficial influence on cognitive processing for written language*” in the first paragraph of the discussion (L. 261).

Second, as addressed earlier, we did not measure the spontaneous occurrence of kūsho behavior in the main experiment; we asked the participants to perform kūsho as they worked in the kūsho condition and we did not allow them to do it in the other two conditions. The significant differences in the number of correct responses of the tasks between the kūsho condition and the other two conditions reflects the facilitative effect of the kūsho behavior on task performance.

Although education years and MMSE scores were not correlated in our data, it is well known that education and cognitive function in older adults are positively correlated, as has already been cited in the manuscript. However, as the link between education years and cognitive functions in older adults may not have been sufficiently emphasized in the previous version of the manuscript, we have added explanations and new citations and modified expressions in the Discussion as follows: “*Education level is known to positively correlate with various cognitive [17, 18, 30, 31] and brain functions [16, 32] in older adults and has also been used as a marker of the concept of cognitive reserve, which refers to susceptibility to age-related changes in cognitive functions [19, 33-35]”* (L. 326) and *“First, kūsho behavior could have clinical and educational benefits for individuals with a decline or a lack of ability in specific cognitive functions such as stroke patients and second-language learners [10]”* (L. 360).

(10) Lines 251–8. You make the case for visual feedback as a prerequisite for the efficacy of kūsho behavior. But how do you deal with the commonly-observed spontaneous use of kūsho in settings where visual feedback is seemingly deliberately unavailable, such as writing on the top of one’s thigh underneath a desk top, or in the air with the writer’s gaze turned away from the kūsho-producing hand? And if visual feedback were necessary to induce the kūsho effect, why would you have to ‘ENCOURAGE [participants] to turn their eyes from the screen to their right index finger’ as opposed to simply relying on their spontaneous integration of visual input to the kūsho effect?

Authors’ response

Regarding the first question, first, one should distinguish between the occurrence of the kūsho action and its practical effect, as discussed in previous studies (Itaguchi et al. 2015, 2017). Second, experimental evidence from the previous study suggests that in the situation that the reviewer referred to, the facilitative effect of kūsho would not be large, at least in terms of psychological experiments (this “effect” refers to a statistical effect size). However, we do not deny the possibility that such behavior undertaken in a place that cannot be seen is helpful in daily life in terms of practical means or brain processing.

Regarding the second question, we controlled the experimental condition to show the facilitative effect of kūsho movements under the same visual condition. If we had not controlled the participants’ gaze, instead relying on participants’ spontaneous watching of the finger movements, it is possible that some participants would never watch their fingers and some participants would occasionally watch their fingers. In such a case, we would not be able to distinguish whether a particular hand condition or the visual feedback of the hand/finger movements is beneficial for the performance.

(11) As conceded in lines 318–9, the sample size is quite small. I would expect that with a larger pool of participants, the effects of age and years of education might be more fully displayed. I would recommend that the study be augmented.

Authors’ response

The number of participants (n = 26) was statistically sufficient to test the expected effect size. As described in the discussion section, the previous study’s effect size was 0.48. We had to detect this effect size but did not want to detect small effect sizes for a fair comparison with previous studies. In multiple comparisons with three comparisons, we found effect sizes of *d* = 0.80 and 0.52, which are large and middle effect sizes, and both of them were statistically significant. When *d* = 0.11, which is a very small effect size, the difference was not statistically significant, “as planned.” With a larger number of participants, it is possible to get meaningless but statistically significant differences for such a small effect size. We believe the number of participants in the current study is appropriate in psychological and experimental studies on human subjects. It is important to keep an adequate sample size (neither too large nor too small) to generalize the results.

There are a few typos, or sites where word choice might be improved.

Lines 22, 25: Missing definite article before ‘__ kūsho effect’

Line 22: Since the participants’ education is not assessed qualitatively or with respect to its content, I would suggest ‘…(MMSE) score, and number of years of education’

Authors’ response

We appreciate your pointing these points out. We modified “the” and “number of years of” (L. 22, L. 24, L. 25).

**Reviewer #2**

1) Abstract: it needs to be rewritten after correcting the manuscript. Specifically the last sentence should be corrected. It cannot be asserted that “The study confirms the benefits of finger movement for “solving cognitive tasks….”. This study deals with the effect of “Kūsho behavior” on identification of written language/kanji characters and not with various cognitive tasks. In addition, it should be stated that: “the kūsho effect may be mediated by “education” and not by “intellectual ability”.

Authors’ response

First and foremost, we greatly appreciate Reviewer #2’s attentive and valuable comments. We agree that the last sentence of the abstract should be modified. Based on the suggestions from the reviewer and those from another reviewer, we have revised the abstract as follows: *“This study confirms the benefits of finger movement for solving cognitive tasks involving visual processing of written language among older adults and suggests the kūsho effect may be mediated by education”* (L. 26)*.*

2) Introduction:
a) My main concern is related to the rationale of assessing the facilitative effect of “Kūsho” in older adults. The authors argue that they wish to test whether the “Kūsho” behavior is universal, regardless of age. However, if this phenomenon occurs due to the written language system, i.e., use of kanji, the phenomenon should be present on every literate person, disregarding the age of the individual. Thus, the authors do not provide a reason for why this phenomenon should not exist in older literate people. I believe that the interest to study older adults in this experimental situation is to evaluate whether “Kūsho behavior” is equally relevant for this population as it is for younger individuals. My understanding is that any literate person in the kanji-system may rely at different degrees on “Kūsho behavior”. If this is true, the reason to assess older persons is to better understand how normal age-related changes in sensorimotor systems and cognitive functions affect “Kūsho behavior”.

Authors’ response

We agree with the reviewer’s comments, and we think it is important to note that the interest of the study was the effect of age-related changes in sensorimotor system and cognitive functions. However, it is also important to note that our focus is not “kūsho behavior” itself but the facilitative effect of kūsho behavior. We therefore revised the expressions as follows: “*The present study, therefore, aimed to investigate whether the facilitative effect of kūsho behavior is observed among kanji-culture older adults as it is among younger adults, regardless of age-related changes in the sensorimotor system and cognitive functions*” in the first paragraph of the introduction (L. 45), “…*assuming the facilitative effect of kūsho behavior exists among older adults…*” in the second paragraph of the introduction (L. 59), and “*…the interaction between language and sensorimotor systems for the facilitative effect of kūsho behavior is still working, at least among kanji-culture older adults*” in the second paragraph of the discussion (L. 277).

b) It would be a strength if the authors add information on “cognitive reserve” (CR), especially in view of their findings on education. CR is particularly important to understand age-related changes, both declines and gains, in late adulthood in any culture and to my knowledge across different languages.

Authors’ response

The reviewer’s suggestion is reasonable and important. Although we could not discuss CR in detail due to a lack of evidence, we have mentioned and cited references in the revised manuscript as follows: “*Education level is known to…and has also been used as a marker of the concept of cognitive reserve, which refers to susceptibility to age-related changes in cognitive functions [19, 33-35]. These previous findings together suggest that the individuals with more years of education in this study might have produced a moderate number of correct responses even in the condition where the kūsho behavior was not available, and, therefore, they benefited less from kūsho behavior than those with shorter education years*” (L. 327).

33.	Stern Y, Gurland B, Tatemichi TK, Tang MX, Wilder D, Mayeux R. Influence of education and occupation on the incidence of Alzheimer's disease. JAMA. 1994;271(13):1004-10.

34.	Piccinin AM, Muniz-Terrera G, Clouston S, Reynolds CA, Thorvaldsson V, Deary IJ, et al. Coordinated analysis of age, sex, and education effects on change in MMSE scores. Journals of Gerontology Series B: Psychological Sciences and Social Sciences. 2012;68(3):374-90.

35.	Soldan A, Pettigrew C, Albert M. Evaluating cognitive reserve through the prism of preclinical Alzheimer disease. Psychiatric Clinics. 2018;41(1):65-77.

c) It would be important to develop more on why vocabulary was not addressed in this investigation. It is well known that vocabulary can be highly important in determining outcomes of language studies and aging. It would be more advisable to actually add vocabulary data to this manuscript. See more comments on this issue below.

Authors’ response

We agree that vocabulary is important, especially for language tasks. We, however, did not report the participants’ vocabulary scores because Japanese older adults, particularly those who are older than 80, tend to show feelings of rejection toward vocabulary tests as they think they were not “educated” because of World War II in their childhood. We actually have vocabulary scores for 12 participants. Although our previous studies revealed no correlation between kūsho effects and vocabulary scores in university students and the kanji characters used were elementary-school level, we do not know the relationship in older populations. Given the reviewer’s suggestion, we added the 12 participants’ data in the supporting data, and added an explanation about it as follows: “*For 12 of the participants, a vocabulary test known as 100 RAKAN [21], which comprises a 100-kanji-word reading, was also conducted. Although we did not consider the vocabulary data in the analysis because of the lack of numbers in the data, the scores for these 12 participants are available in the Supporting Information*” (L. 95).

3) Methods:
A) It seems that the MMSE cut-off of 24 was not use as inclusion or exclusion criteria, which is understandable due to the advanced age of many of the participants. However, the authors claim to have examined a sample of healthy older adults. This cannot be stated since there are participants scoring below MMSE 24 who might have important cognitive declines possibly related to dementia or mild cognitive decline. This issue needs to be addressed and acknowledged.

Authors’ response

Based on the reviewer’s suggestion, we have removed “healthy” from the abstract, introduction, and discussion in the revised manuscript. In the methods section, we added an explanation, as follows: “*For two participants, the MMSE scores were below 24, which is the standard cut-off value for healthy and mild cognitive decline*” (L. 89).

B) It would strength the study to present data on vocabulary in case that such data are available. It would be important to show whether education, vocabulary, or both exert an effect on less deployment of Kūsho behavior in older adults.

Authors’ response

Yes, we agree with the reviewer’s suggestion. As addressed above, we only have such data for 12 participants, and the data are available in the Supporting Information section.

C) Why is handedness measured and reported? The authors do not mentioned this variable in either the introduction or the discussion. If it is no of importance, it should be removed.

Authors’ response

The reviewer’s suggestion is reasonable, because we do not assume that handedness is important for the kūsho effects. However, in language-related studies, we think that handedness is important and should be described or controlled, since we know that there is a difference in lateralization of brain function. We therefore are keeping this information in the manuscript for future reference.

4) Results:

a. Related to the previous comment, it would be interesting to compare the relative importance of education vs. vocabulary. Findings would enhance the interest of the study.

Authors’ response

As addressed above, we do not have sufficient data for a fair analysis. Just for reference, we calculated a correlation matrix (not partial correlations) including vocabulary score using data from 12 participants. Correlation coefficients among age, education, and MMSE were largely different between the two calculations (*n* = 26 vs. *n* = 12). Vocabulary is correlated more with age than with education, but the values may be unstable when *n* = 12 (Table s2).

Table s1. Correlation matrix (*n* = 26). These coefficients are described in Table 2 in the manuscript.

|  | Education | MMSE |
| --- | --- | --- |
| Age | 0.28 | 0.03 |
| Education |  | 0.15 |

Table s2. Correlation matrix (*n* = 12)

|  | Education | Vocabulary | MMSE |
| --- | --- | --- | --- |
| Age | 0.68 | 0.79 | 0.22 |
| Education |  | 0.52 | 0.46 |
| Vocabulary | |  | 0.04 |

5) Discussion:

a. If vocabulary data were available, it would be necessary to expand the discussion on p. 11 to cover “Educational level vs vocabulary level and Kūsho effect”.

Authors’ response

Again, we are sorry that, as addressed above, we do not have sufficient data for discussion. We have added the possibility that vocabulary size is involved in the kūsho effect, as follows: “*Another candidate may be vocabulary size; vocabulary scores are known to be positively correlated with education years [37-39]. Although vocabulary score has been suggested as not being associated with the kūsho effect in younger adults [10], there is still a possibility that it interacts with the sensorimotor processing in older populations*” (L. 399).

b. This part should also be amended by including a discussion on CR and the final findings that in the best of cases comprise both education and vocabulary.

Authors’ response

As addressed earlier, we have added a discussion on CR as well as citations, as follows: “*Education level is…and has also been used as a marker of the concept of cognitive reserve, which refers to susceptibility to age-related changes in cognitive functions [19, 33-35]. These previous findings together suggest that the individuals with more years of education in this study might have produced a moderate number of correct responses even in the condition where the kūsho behavior was not available, and, therefore, they benefited less from kūsho behavior than those with shorter education years* ” (L. 327).

33.	Stern Y, Gurland B, Tatemichi TK, Tang MX, Wilder D, Mayeux R. Influence of education and occupation on the incidence of Alzheimer's disease. JAMA. 1994;271(13):1004-10.

34.	Piccinin AM, Muniz-Terrera G, Clouston S, Reynolds CA, Thorvaldsson V, Deary IJ, et al. Coordinated analysis of age, sex, and education effects on change in MMSE scores. Journals of Gerontology Series B: Psychological Sciences and Social Sciences. 2012;68(3):374-90.

35.	Soldan A, Pettigrew C, Albert M. Evaluating cognitive reserve through the prism of preclinical Alzheimer disease. Psychiatric Clinics. 2018;41(1):65-77.

**Reviewer #3:** Authors have conducted a series of experiments investigating kusho behaviors. Now, they extend their target to elderly people living in Japan. I appreciate their new contribution using elderly people. However, their research question is not clearly written in the current manuscript. For that reason, I suppose, their discussion also has not been deepened sufficiently enough.

I would point out two main negatives of this paper:

(i) No comparisons with younger participants

(ii) No further examinations of cognitive abilities specifically, memory.

I suspect that these negatives might come from the fact that they had no valid hypothesis when they started this study. To compensate these negatives, authors should work even harder. I will try to provide them inspirations for that as follows.

**Major points**

(1) Motivation of the study.

I have to say that authors did not successfully explain their motivation to study elderly people. I know they stated:

While kūsho behavior is widely observed among Japanese people regardless of age, no previous study has specifically confirmed the effect of kūsho behavior on cognitive processing among older adults. The present study, therefore, aimed to investigate whether the facilitative effect of kūsho behavior is universal, regardless of age, at least among kanji-culture adults. (lines 41-44)

This does not sound intrinsic. I would like authors to more clearly ensure accountability for the employment of elderly people for this study.

I will give some suggestions regarding this point (although I do not mean to force authors to discuss these; they would achieve better than me.)

(i) What is the main influence of the aging on kusho behaviors? No effects? Reducing or amplifying?

(ii) The computer environment of elderly people when they learned kanji was greatly different from the modern one. In a sense, they evaded the use of electric devices during their childhood. Then, the kusho effects might be genuinely observed among them as compared to younger participants.

(iii) In spite of the memory decline generally seen among the elderly, the kusho movements would be stably maintained. Even more, it may facilitate the memory of kanji.

Authors’ response

First of all, we greatly appreciate Reviewer #3’s extensive and valuable comments. The motivation of the analysis of elderly people’s behavior was to investigate the effects of age-related changes in sensorimotor system and cognitive functions. Given the suggestion from the reviewer and another reviewer, we have revised the expressions as follows: ““The present study, therefore, aimed to investigate whether the facilitative effect of kūsho behavior is observed among kanji-culture older adults as it is among younger adults, regardless of age-related changes in the sensorimotor system and cognitive functions” in the first paragraph of the introduction (L. 45), “…*assuming the facilitative effect of kūsho behavior exists among older adults…*” in the second paragraph of the introduction (L. 59), and “*…the interaction between language and sensorimotor systems for the facilitative effect of kūsho behavior is still working, at least among kanji-culture older adults*” in the second paragraph of the discussion (L. 277).

(2) Dementia and MCI

It was good that authors examined MMSE. I found by Table 1 and Figure 3 that they included participants who were categorized as MCI or even as dementia (score 21). Authors should discuss the effects of MCI/dementia on kusho.

Authors’ response

First, considering this reviewer’s and another reviewer’s suggestion, we have removed “healthy” in the abstract, introduction, and discussion in the revised manuscript. Second, in the methods section, we added explanation as follows: “*For two participants, the MMSE scores were below 24, which is the standard cut-off value for healthy and mild cognitive decline*” (L. 89). Furthermore, given the reviewer’s comment, we have added a discussion about MMSE as follows: “*…specific, but not general, cognitive declines are likely to exhibit a larger kūsho effect*” (L. 325) and “*In the present study, MMSE scores, which reflect general cognitive function, was not related with education or the kūsho effect, suggesting that the increase in kūsho effect was not attributed to general cognitive decline, but to a decline in rather specific cognitive functions*” (L. 332). Last, in the final paragraph of the discussion, we refer to MCI patients as follows: *“Accordingly, although kūsho behavior could be beneficial for individuals with mild cognitive impairments, it may not correlate with MMSE score”* (L. 367).

(3) ANCOVA

I think they could use ANCOVA instead of ANOVA for their analysis, including age, MMSE score and years of education as the covariates.

Authors’ response

We used a within-subject ANOVA, but not a between-subject ANOVA, to detect the effect of hand conditions. If we had used a between-subject ANOVA, as the reviewer pointed out, ANCOVA would be required to control confounding variables among different groups. However, in a within-subject ANOVA, the effects of confounding variables equally influence all hand conditions, and, therefore, ANCOVA is not necessary in our statistical design.

Even when an ANCOVA is conducted on the data with the covariates of age, MMSE score, and education years, the result is not changed (*F* (2, 48) = 6.18, *p* < .01) from that of the ANOVA (*F* (2, 50) = 7.22, *p* < .01). Note that the degree of freedom differed from that in the ANOVA because we had one missing piece of data for the MMSE, and, therefore, the F and p values slightly differed from those of the ANOVA.

(4) Paragraph writing

I would suggest that authors rewrite all paragraphs to ensure that a conclusion statement comes to the first line and then the explanations follow in the rest of the paragraph.

Authors’ response

We have modified several expressions and the order of sentences in the second and third paragraphs in the introduction and the fifth and sixth paragraphs in the discussion.

**Minor points**

(5) Lines 36-37, “kanji writing-system users”: this expression is inappropriate because Chinese speaking people do not call their character kanji. Instead, authors may just want to use “character users” or “people who use a writing system with characters”.

Authors’ response

We agree with the reviewer’s point and have changed the expression as follows: “…*among people who use a Chinese character writing system (kanji)…*” (L. 38).

(6) Participants: did all participants prefer to use their right hand for writing? Authors should clearly state this point.

Authors’ response

All participants’ preferred hand for writing was the right hand. We have added this explanation, as follow: “*All participants preferred to use their right hand for writing…*” (L. 82).

(7) Experimental settings: how far was the computer from a participant? How many experimenters stayed beside a participant and what locations? Authors should explain how they observed participants’ responses sufficiently.

Authors’ response

The computer display was away about 50 cm away from the participants. Two experimenters observed participant’s gaze (one from the side and the other from the front of the participant). Participants were instructed to face their hand/fingers, not just move their eyes, during the thinking process. Therefore, the judgment of whether the participants followed the instruction was obvious and easy. We have added/modified the explanation as follows: “*…on a computer display [6, 9, 10], which was about 50 cm away from the participants*” (L. 104), and “…*participants were instructed to turn their face from the screen and watch their right index finger or hand in a trial…*” (L. 145).

(8) Stimuli: how did they decide the locations of subparts in the triangle? Did authors avoid a subpart to write first in the original kanji came to the top of the triangle or never mind and put it randomly?

Authors’ response

Following the method used in a previous study (Itaguchi et al. 2017), the arrangement of sub-parts was defined to have a relative relation that was different from the original; they were not randomly arranged. Since we used only three sub-parts, there were few alternatives for the arrangement. We have added the explanation in the method section as follows: *“The sub-parts were placed in a unique arrangement, different from the original one…”* (L. 168).
