# Supplementary material for: Writing in the air: Facilitative effects of finger writing in older adults
Source: PLoS One. 2019 Dec 27;14(12):e0226832. doi: 10.1371/journal.pone.0226832 (PMC6934278; doi:10.1371/journal.pone.0226832)
Supplement: Attachment — Submitted filename: Responses_to_reviewers1003.docx [file pone.0226832.s003.docx]

Dear Editor and Reviewers,

We greatly appreciate your rigorous and fair review of the manuscript as well as your insightful comments. The revised manuscript has been modified in response to the comments, as follows:

All comments from the reviewers have been responded to in the following pages. In the revised manuscript, revisions are highlighted using violet font (red is for the first revision).

We look forward to hearing from you regarding our submission. We would be glad to respond to any further questions and comments that you may have.

Sincerely,

Yoshihiro Itaguchi

Responses to Reviewer 1’s comments	p. 2

Responses to Reviewer 2’s comments	p. 4

Responses to Reviewer 3’s comments	p. 6

\# Reviewer 1

First of all, we greatly appreciate Reviewer 1’s attentive and valuable comments. We have considered the suggestion from Reviewer 1 deeply, and would like to explain our point of view in detail.

(1) The revisions of this ms. raise its quality, but feel a bit superficial. My objections number (10) is smoothed over without really resolving it; (11) is sidestepped;

Authors’ response:

In the number (10) of the previous review, the reviewer raised two concerns:

(1) *But how do you deal with the commonly-observed spontaneous use of kūsho in settings where visual feedback is seemingly deliberately unavailable, such as writing on the top of one’s thigh underneath a desk top, or in the air with the writer’s gaze turned away from the kūsho-producing hand?*
(2) *And if visual feedback were necessary to induce the kūsho effect, why would you have to ‘ENCOURAGE [participants] to turn their eyes from the screen to their right index finger’ as opposed to simply relying on their spontaneous integration of visual input to the kūsho effect?*

First, in the present study, we did not examine the situation the reviewer pointed out, and thus we cannot refer to the situation as “*the commonly-observed spontaneous use of kūsho in settings where visual feedback is seemingly deliberately unavailable.”* Furthermore, we do not have any evidence regarding how frequently we use kūsho without its visual feedback. As we have answered in the previous response letter, and described in the manuscript, previous studies did not find statistically significant facilitative effects of kūsho behavior without its visual feedback, at least in the kanji construction task. We do agree that the situation the reviewer pointed out is important to understand the mechanism underlying the interaction of the finger movements and cognitive processing. However, we think that the content of the comment number (1) is beyond the scope of the current study.

Second, as previously answered in the previous response letter and also described in the manuscript, we controlled the experimental condition to test the facilitative effect of “kūsho movements” under the same visual condition. This is the only reason why we had participants watch their hand or finger movements. However, it is possible that we have not fully understood the reviewer. We would greatly appreciate if the reviewer clarifies the meaning of the question and shows how we could examine the facilitative effect of “kūsho movements” under uncontrolled visual conditions that depend on participants’ changeable will.

Regarding comment (10), we are not sure whether the reviewer agrees or disagrees with the explanation in terms of the statistical hypothesis testing. If the reviewer disagrees with the explanation in terms of the statistical testing, it would be extremely helpful if the reason is explained in greater detail. According to our understanding, it is important to have a sample of adequate size to generalize the results (the details have been already explained in the previous letter); therefore we do not need larger samples to test the hypothesis.

(2) I don't think the authors have really dealt with a matter raised by Reviewers #2 and #3, "No comparison with a younger cohort".

Authors’ response:

As you have indicated, reviewer 3 did point out that the lack of comparison with younger adults is one of the limitations of our study (reviewer 2 did not refer to this point); however, reviewer 3 did not require us to directly “deal with” the limitation. Although we agree that it would have been better if populations of different ages had been compared, the lack of the comparison in the study is not necessarily a weakness of this study. One of the reasons is that we conducted an experiment using the same procedure, program, and stimuli with those used in a previous study for younger adults (Itaguchi et al. 2017) and directly compared the effect sizes of the facilitation effect of kūsho between the studies in the discussion section. To emphasize this point, we have added the sentence *“Note that the experiment procedure, program, and stimuli were the same between the study for younger adults [10] and the current study”* in the discussion of the revised manuscript. The purpose of the present study is to show the existence of the facilitation of and not the difference of the kūsho effect between the two populations.

\# Reviewer 2

First and foremost, we greatly appreciate Reviewer 2’s attentive and valuable comments.

(1) In the abstract, I suggest to better clarify the purpose of the investigation. Instead of: “This study investigated whether kusho could help older adults solve a cognitive task” it should be: “This study investigates whether the facilitative effect of kusho is affected by old age”."

Authors’ response

We agree with Reviewer 2’s suggestion and have modified the expression of the purpose of the study as follows: "*This study investigates whether the facilitative effect of kūsho, the existence of which has been exclusively confirmed in younger adults, is affected in old age*" (L20)

(2) Supporting information: I understand that due to the missing data on vocabulary the authors are not able to address the role played by this variable and therefore they provided few existing results as supplementary material. Though, they have to explain the sense of these data. In other words, whether higher scores reflect higher lexical skills.

Authors’ response

We have added explanations about the test as follows: “100 RAKAN [21], which comprises a 100-kanji-word reading, *standardized based on visual and phonetic aspects of word familiarity,* was also conducted *to evaluate their vocabulary size*. *The minimum and maximum scores of the test are 0 and 100, respectively.”* (L96)

(3) Discussion p. 12:
    a. The first sentence on the 2nd paragraph needs to be rephrased (lines 324 to 326). The meaning is not clear to me. “Although this study did not clarify the exact factors and mechanisms that depend on education years, specific, but not general, cognitive declines should intervene a larger kūsho effect.”
    b. Lines 334 and 335: It should be “global cognitive status” and not “general cognitive decline”.
    c. Line 335: The authors claim that a possible candidate mediating the effects of education is visual processing. However, it is largely known that older adults suffer normal declines not only on their visual acuity, but also on their perceptual visual abilities (see e.g., Monge & Madden, 2016). Importantly, no visual evaluation was conducted in this study. Thus, I would rather suggest thinking about the advantages of well-learned proprioceptive functions in this population. Some authors have suggested that no age effect exists in proprioception when older adults know in advance the movements related to a specific action (Stelmach & Sirica, 1986).

Authors’ response

(a) To clarify the meaning of the sentence, we have removed “but not general” and modified the sentence as follows: “*Specific cognitive declines are likely to intervene in a larger kūsho effect, although this study did not clarify the exact factors and mechanisms that are modulated by years of education.”* (L328)
(b) We have corrected the term into “global cognitive status” as the reviewer suggested. Thank you for pointing it out. (L338)
(c) First, we agree that older adults suffer declines on visual acuity and perceptual visual abilities. In the previous manuscript, we mentioned “visual processing,” which originally referred only to higher-level, and not lower-level visual functions. To clarify this, we have added “*higher-level*” in the revised manuscript. (340)

Second, we do not think that “relatively preserved proprioceptive function” in older adults contributed to the facilitative effect of kūsho movements, as previous findings that kūsho movement without visual feedback, where participants had to rely on their proprioception, did not facilitate the task performance. However, as we did not directly test the effect of visual feedback on the kūsho effect, there is still a possibility that proprioceptive feedback of the kūsho movements also contributed to the facilitative effect, as the reviewer suggested. Therefore, we have added a possibility of the contribution of kinesthetic feedback on the task performance as follows: “*Kinesthetic feedback of kūsho finger movement, which was shown to have only little (non-significant) effect on task performance if the visual feedback of the movement is not available in younger adults [9, 10], could also contribute to the facilitation effect in older adults, as the importance of visual feedback in the task processing was not directly tested in the present study.*” (L347)

\# Reviewer 3

We greatly appreciate Reviewer 3’s attention to detail and valuable comments.

(1) I recognized that the authors improved the manuscript greatly. However, I still have an uncomfortable feeling against their logics. I will indicate two points; their aim of the study and their way of description.

Their aim of the study:

First, I would categorize two types of papers.

(1) Papers that reveal a new phenomenon.

(2) Papers that investigate a mechanism of a phenomenon.

This paper seems to belong to the first one, with having a factor of the second. It revealed that the elderly people in Japan showed kusho behaviors in a similar way as having shown in younger generations, and as well investigated the relationship with individual parameters including years of education.

If my understanding above is correct, then I suppose their main aim of the study was to reveal the incidence of kusho behaviors among elderly people in Japan. The authors might originally have no interests in the mechanism of that. However, they happened to find a significant difference in years of education, which was included in questionnaire entries to obtain the basic information of subjects. By this observation, they found themselves to be obliged to discuss that in the paper.

Am I right?

If it was the case, I would like authors to more clearly indicate these backgrounds. Otherwise, I feel a difficulty to get their points in the current version of the manuscript.

Also, I would like authors to more explicitly explain why they considered it was important to reveal the incidence of kusho among the elderly in Japan. I suppose this was the main claim that authors should have appealed.

Authors’ response

Thank you for clarifying the reviewer’s understanding of the manuscript. However, there seems to be a misunderstanding. The purpose of the study was to confirm *the existence of the facilitative effect* of kūsho behavior in older adults, as described in the manuscript, and NOT to show the *“incidence of kusho behaviors among elderly people in Japan.”* Based on previous studies, we originally assumed information processing to impact the facilitative effect and thus planned to dissociate the effects of several types of kinesthetic feedback on the kūsho behavior. Furthermore, in this study, to understand the interaction between finger movement and cognitive processing in greater detail, we planned to explore the important factors related to the facilitative effect, by considering more diverse demographic factors of older adults and comparing them to young adults. This background has already been described in the manuscript.

(2) Actually, I think it might be better if they had an interest in the investigation of the mechanism of kusho from the beginning. They might want to measure “sensorimotor system” (line 47) possibly using RT of a button press to a visual cue and “cognitive functions” (lines 47-48) using assessments. They tried to use 100 RAKAN (line 95) for the latter, but unfortunately, it was not completed to all participants.

Here, for the next chance of the continuation of this study, another idea would be Japanese Adult Reading Test (JART), which was standardized and relatively easy to conduct."

Authors’ response

We are extremely grateful for your suggestion for future studies. JART, which comprises 50 kanji readings, is a test for the evaluation of IQ, which is a very complex index; however, it is not a test for any specific cognitive function or skill. Although we assume that its scores would correlate with the task performance, we are afraid that we would not be able to draw significant conclusions from the data. Furthermore, the problem related to the use of kanji reading tests in Japanese older adults are not solved because JART uses kanji reading like 100 RAKAN. Nevertheless, it is a very significant suggestion and we thank you for it.

(3) Their way of description:

Another reason that I feel a difficulty in grasping their points is their way of writing the manuscript. I will raise two points: paragraph writing (again) and reduction of sentences beginning with a particle.

(1) Paragraph writing

As I wrote in the previous review, the authors frequently put their conclusion (main statement) at the last part of the paragraph. This is a feature that is frequently observed among people from Asia. I would suggest that they can move the last sentence to the top of the paragraph.

Example #1: page 3, the paragraph beginning with “To investigate…” (line 49)

They may want to start the paragraph with the last sentence of this paragraph, i.e., “In the present study…” (lines 58 - 61). They may want to modify it such as:

We hypothesized that the elderly people would show a better cognitive performance in a kusho condition than in the other conditions because… something like this.

Example #2: page 10, the paragraph beginning with “While previous studies…” (line 266)

They may want to start with the last sentence of this paragraph, i.e., “The present results suggest that the interaction between…” (Lines 277 – 279).

Then, they could deepen and strengthen the discussion.

Example #3: page 12, the paragraph beginning with “Although this study did not clarify…” (line 324)

They may want to start with the last sentence of this paragraph, i.e., “Our data suggest that decline in a specific function may be involved with a modulation of the kūsho effect in the kanji construction task” (lines 343-344)."

(2) Reduction of sentences beginning with a particle

There are too many sentences beginning with a particle (e.g., For…, In the…) . Please reduce them by half.

By the way, did authors ask a copy-editing company? If not, they should ask.

Authors’ response

We greatly appreciate the reviewer’s views and explaining them in detail using suitable examples and solutions. We understand the reviewer’s policy (“concluding first”), but we think the current versions of first sentences of the paragraphs introduce the “topic” of the paragraph, instead of specific conclusions. In our understanding, the first sentence (topic sentence) of a paragraph should outline the main idea of the paragraph, which need not necessarily indicate the specific conclusion of the paragraph.

e.g., https://www.sheffield.ac.uk/ssid/301/study-skills/writing/academic-writing/paragraph-flow-connectivity;

https://owl.purdue.edu/engagement/ged_preparation/part_1_lessons_1_4/index.html;

https://wts.indiana.edu/writing-guides/paragraphs-and-topic-sentences.html;

https://www.englishbooks.jp/catalog/WB1-U4.pdf

Regarding Example #1, both the first and last sentence of the paragraph refer to the same idea. However, the first sentence presents it in a rather simple and abstract way, and the last sentence presents it in a more concrete way with further details. It is the same for Examples #2 and 3; we first outline the contents of the paragraph (i.e., “our hypothesis was supported,” or “specific cognitive declines are likely to intervene in a kūsho effect”), and the last sentence states it again with more concrete details. We hope this style is acceptable.

Following the reviewer’s suggestion, we have modified the first sentences with a particle (L49, L148, L270, and L328).

The manuscript has been edited by an editing company (Editage).

Minor points.

(1) Please remove discussion from Materials and Methods.

Authors’ response

We are not sure as to which part the reviewer is referring to. We would be extremely grateful if you could please clarify it using a line number.

(2) Line 153. What did they mean by “only one visual condition”? My understanding is that authors had 3 conditions, as shown in Figure 2. In lines 349-350, they also wrote; “this study used only one visual condition instead of comparing the two visual conditions.” Please clarify what was “one” or “two”.

Authors’ response

We have added “*, using two visual conditions (eye-on-hand and eye-on-display conditions),*” and “if one does not see the finger movement *(eye-on-display condition),*” and modified “Only one” into “Only *the eye-on-hand*” to clarify the visual condition we used, in line with previous studies (Itaguchi et al. 2017; Itaguchi et al. 2015).

(3) Authors frequently use the word “hand condition”. Could it be just “experimental condition,” because there were no “foot” conditions?

Authors’ response

We used the term to ensure consistency with the term used in previous studies (Itaguchi et al. 2017; Itaguchi et al. 2015), that is, “hand condition.” We think the term “Experimental condition” is not appropriate here because the meaning is vague with regard to experimental manipulation.

(4) I found a discrepancy in the description of RT. At line 121, they wrote; “Response times were not statistically analyzed or discussed because participants’ voices were not always loud or clear enough to detect the onset of the responses, and the ratio of missing values was very high (31% of correct responses were missing values).” However, they reported RT in line 209; “The average response times were 6.9 s (SD = 1.2) in the kūsho condition, 6.5 s (SD = 1.8) in the static condition, and 6.8 s (SD = 1.3) in the circle-drawing condition.” Did they mean that the report was for the remaining 69% (i.e., 100 – 31) of responses? Also, how did they measure RT? Please clarify.

Authors’ response

We are not sure if the reviewer is referring to a discrepancy in the usage of the term “*statistically analyzed.*” Assuming that our understanding is correct, we modified the expression “*statistically analyzed*” into “*statistically tested.*”

Yes, the remaining 69% was used to calculate the RTs. We have added “*and 69% of the data were used to calculate response times*.”

As already described in the manuscript, verbal responses were recorded using a microphone. We have added “*and calculate response time.*”
