# Supplementary material for: Writing in the air: Facilitative effects of finger writing in older adults
Source: PLoS One. 2019 Dec 27;14(12):e0226832. doi: 10.1371/journal.pone.0226832 (PMC6934278; doi:10.1371/journal.pone.0226832)
Supplement: Attachment — Submitted filename: PONE-D-19-16768 R2 Comments.docx [file pone.0226832.s004.docx]

Lines 20–22

*This study investigates whether the facilitative effect of kūsho, the existence of which has been exclusively confirmed in younger adults, is affected in old age.*

Does this make sense: *‘the facilitative effect of kūsho… is affected in old age’* ? Is this text a study of whether that ‘*effect’* is ‘*affected’*?

Rather, don’t you mean:

*This study investigates whether the facilitative effect of kūsho, the existence of which has been exclusively confirmed in younger adults, is present in old age.*

Line 99

*because of lack of numbers in the data*

I’m not sure what this means. Numbers of…what?

Apparently, you did record the participants’ scores on the RAKAN; what is the point of mentioning that you had them take this test if their scores are not worth reporting in the body of your text?

Line 125

To make this a bit clearer, I’d suggest that you specify *31% of correct responses were missing values for response time*

Lines 256*ff*

I feel that the Discussion section is significantly strengthened in this version of the ms.

Lines 335–336

Instead of :

*they benefited less from kūsho behavior than those with shorter education years*

I would suggest:

*they benefited less from kūsho behavior than those with fewer years of education*

Lines 338–9

Remove ‘*the*’

*not attributed to global cognitive status*

Line 340

Remove hyphen:

*to explain this could be the high level of visual processing*

Lines 348–350

Remove ‘*the’* x 3

*effect on task performance if visual feedback of movement is not available in younger adults …the importance of visual processing in task processing*

Lines 357, 359

Remove ‘*the’* x 2

*it has been shown that visual feedback of finger movement*

*instead of comparing two visual conditions*

Lines 373–374

ADD ‘*the’;* use ‘*of’* genitive

*Our finding that the kūsho effect was negatively related to years of education*

Lines 377–378

Instead of:

*can provide another suggestion for embodied cognition.*

I would suggest:

*may provide an instantiation of embodied cognition*

or

*may serve as an additional example of embodied cognition.*
