# Supplementary material for: Writing in the air: Facilitative effects of finger writing in older adults
Source: PLoS One. 2019 Dec 27;14(12):e0226832. doi: 10.1371/journal.pone.0226832 (PMC6934278; doi:10.1371/journal.pone.0226832)
Supplement: Attachment — Submitted filename: Kusho_older_adults_20191203_Responses.docx [file pone.0226832.s005.docx]

Dear Editor and Reviewers,

We greatly appreciate your rigorous and fair review of the manuscript as well as your insightful comments. The revised manuscript has been modified in response to the reviewers’ comments.

In the revised manuscript, revisions are highlighted using blue font (red and violet are for the previous revisions).

We look forward to hearing from you regarding our submission.

Sincerely,

Yoshihiro Itaguchi

12/03/2019

Responses to Reviewer 1’s comments	p. 2

Responses to Reviewer 2’s comments	none

Responses to Reviewer 3’s comments	p. 4

Reviewer #1:

First, we greatly appreciate Reviewer #1’s detailed checking and conscientious indications. Reviewer #1’s suggestions are important for improvement of clarity of the manuscript. Following the suggestions, we have modified the manuscript:

(1) Lines 20–22

*This study investigates whether the facilitative effect of kūsho, the existence of which has been exclusively confirmed in younger adults, is affected in old age.*

Does this make sense: *‘the facilitative effect of kūsho… is affected in old age’* ? Is this text a study of whether that ‘*effect’* is ‘*affected’*?

Rather, don’t you mean:

*This study investigates whether the facilitative effect of kūsho, the existence of which has been exclusively confirmed in younger adults, is present in old age.*

Authors’ response:

Thank you for pointing out. We agree with the suggestion and have corrected the word to “present”.

(2) Line 99

*because of lack of numbers in the data*

I’m not sure what this means. Numbers of…what?

Apparently, you did record the participants’ scores on the RAKAN; what is the point of mentioning that you had them take this test if their scores are not worth reporting in the body of your text?

Authors’ response:

As the reviewer pointed, the sentence was weird. We have corrected the sentence to *“because several participants did not perform the test”* (L99). We did not mention the scores because the number of the data was not equal to that of the other indices (therefore their average value may be biased), we did not analyze them, and also a previous study showed that vocabulary score was not correlated with the amplitude of kusho effect in younger participants. We, however, agreed with the suggestion by the reviewer #2 that it is important to report their vocabulary scores, and therefore individual data of the vocabulary score are provided as supporting information. Although the detailed scores do not appear in the main manuscript, a possible interaction between vocabulary score and kusho effect is discussed in Discussion.

(3) – (10)

Authors’ response:

Thank you for your detailed checking. We have modified all the expressions that the reviewer #1’ pointed out (Ls 126, 336, 338, 340, 348, 357, 359, 372, and 376).

(3) Line 125

To make this a bit clearer, I’d suggest that you specify *31% of correct responses were missing values for response time*

(4) Lines 335–336

Instead of :

*they benefited less from kūsho behavior than those with shorter education years*

I would suggest:

*they benefited less from kūsho behavior than those with fewer years of education*

(5) Lines 338–9

Remove ‘*the*’

*not attributed to global cognitive status*

(6) Line 340

Remove hyphen:

*to explain this could be the high level of visual processing*

(7) Lines 348–350

Remove ‘*the’* x 3

*effect on task performance if visual feedback of movement is not available in younger adults …the importance of visual processing in task processing*

(8) Lines 357, 359

Remove ‘*the’* x 2

*it has been shown that visual feedback of finger movement*

*instead of comparing two visual conditions*

(9) Lines 373–374

ADD ‘*the’;* use ‘*of’* genitive

*Our finding that the kūsho effect was negatively related to years of education*

(10) Lines 377–378

Instead of:

*can provide another suggestion for embodied cognition.*

I would suggest:

*may provide an instantiation of embodied cognition*

or

*may serve as an additional example of embodied cognition.*

Reviewer #3:

We greatly appreciate Reviewer #3’s patient reviewing and thoughtful advice. Reviewer #3’s suggestions are important for improvement of the importance of the study in the field.

(1) Page 5, lines 116-118.

In Fig 1a, for example, to make the original kanji character, all subparts were vertically compressed while the top subpart was placed such that it overlapped with the other parts.

The example in the Fig. 1a does not appear to fit to the explanation; the explanation also does not match to the caption of Fig. 1.

Authors’ response:

Thank you for pointing it out. The part was completely our fault and an explanation for another stimulus. We have corrected the explanation as follows: *In Fig 1a, for example, to make the original kanji character, one subpart was placed on the left side and horizontally compressed, and the other subparts were vertically compressed and arranged on the right side* (L117)*.*
